# Potential Utility of Combined Presepsin and LDH Tracking for Predicting Therapeutic Efficacy of Steroid Pulse Therapy in Acute Exacerbation of Interstitial Lung Diseases: A Pilot Study

**DOI:** 10.3390/jcm14093068

**Published:** 2025-04-29

**Authors:** Yuichiro Takeshita, Yasuo To, Masako To, Naho Furusho, Yusuke Kurosawa, Toru Kinouchi, Mitsuhiro Abe, Jiro Terada, Yuji Tada, Seiichiro Sakao

**Affiliations:** 1 Department of Pulmonary Medicine, International University of Health and Welfare Narita Hospital, 852 Hatakeda, Narita 286-8520, Chiba, Japan; y.to@iuhw.ac.jp (Y.T.); furusho.naho@nihon-u.ac.jp (N.F.); yusuke.kurosawa0813@gmail.com (Y.K.); tkinouchi@iuhw.ac.jp (T.K.); ytada25@iuhw.ac.jp (Y.T.); sakaos@iuhw.ac.jp (S.S.); 2 Department of Respiratory Medicine, Japanese Red Cross Narita Hospital, 90-1, Iida-cho, Narita 286-8523, Chiba, Japan; mthrsgnm@yahoo.co.jp (M.A.); jirotera@naritasekijyuji.jp (J.T.); 3 Department of Laboratory Medicine, Dokkyo Medical University, Saitama Medical Center, 2-1-50 Minami-Koshigaya, Koshigaya 343-8555, Saitama, Japan; m-to@dokkyomed.ac.jp; 4 Division of Respiratory Medicine, Department of Internal Medicine, School of Medicine, Nihon University, 30-1, Oyaguchi-Kamichou, Itabashiku, Tokyo 173-8610, Japan

**Keywords:** acute exacerbation of interstitial lung disease, presepsin, lactate dehydrogenase, steroids, therapeutic efficacy

## Abstract

**Background/Objectives**: The usefulness of presepsin, which is released from macrophages, in acute exacerbation of interstitial lung diseases (AE-ILDs) is unknown. We aimed to investigate the utility of monitoring presepsin with other AE-ILD markers before and after steroid pulse therapy in AE-ILDs. **Methods**: This pilot single-center retrospective observational study involved 16 patients with AE-ILDs, including the AE of idiopathic pulmonary fibrosis and idiopathic nonspecific interstitial pneumonia and rapidly progressive connective tissue disease-associated ILD. Patients who survived 90 days were assigned to the survival group (*n* = 9). The remaining patients were classified in the non-survivor group (*n* = 7). To evaluate the therapeutic efficacy of steroid pulse therapy, specific serum markers were selected—presepsin, as a novel AE-ILD marker, and surfactant protein D, C-reactive protein, and lactate dehydrogenase (LDH), as classical AE-ILD markers. **Results**: Thirteen out of sixteen patients with AE-ILDs showed high presepsin levels (presepsin ≥ 470 pg/mL) before steroid pulse therapy. The post-/pre-presepsin ratio and the post-/pre-LDH ratio, calculated by dividing the presepsin and LDH levels after therapy by the levels before therapy, respectively, showed a positive correlation (r = 0.579, *p* = 0.021). As a result of this correlation, the post-/pre-presepsin–LDH index was created, obtained from the “post-/pre-presepsin ratio” multiplied by the “post-/pre-LDH ratio”. In a receiver operating characteristic curve analysis for non-survival, the post-/pre-presepsin–LDH index showed good discrimination as a prognostic marker for a poor outcome (AUC: 0.873, 95% confidence interval: 0.655–0.999). **Conclusions**: Tracking presepsin and LDH simultaneously may be useful for determining treatment response to steroid pulse therapy in the clinical management of AE-ILDs.

## 1. Introduction

Interstitial lung diseases (ILDs), including idiopathic pulmonary fibrosis (IPF), are a group of respiratory diseases that can show poor prognostic outcomes when acute exacerbations occur [1,2]. Clinically, if a chest X-ray or computed tomography (CT) scan shows bilateral ground-glass opacity or consolidation accompanied by acute respiratory failure (ARF), an acute exacerbation of ILDs (AE-ILDs), including an acute exacerbation of IPF (AE-IPF), should be considered as one of the underlying conditions [3,4]. IPF, as well as fibrotic interstitial lung diseases other than IPF, such as nonspecific interstitial pneumonia (NSIP), chronic hypersensitivity pneumonitis (CHP), and connective tissue disease-associated interstitial lung disease (CTD–ILD), are known to cause acute exacerbation (AE) [5]. Although there is insufficient evidence regarding the treatment of AE-ILDs, corticosteroids are the standard for patients with AE-ILDs with rapidly worsening clinical symptoms [6]. In the Japanese guidelines, treatment with high-dose corticosteroids, including steroid pulse therapy, is weakly recommended owing to the high mortality rate associated with AE-IPF [7]. While corticosteroids are commonly used, recent evidence suggests that they may not improve survival in all AE-IPF cases [8]. Therefore, there is an urgent need to identify serum markers that reflect an early response to steroid pulse therapy in patients with AE-ILDs.

Several previous studies have suggested that AE-ILDs are involved in macrophage-mediated inflammation; therefore, it is important in clinical practice to follow biomarkers that sensitively reflect macrophage activity in AE-ILDs after steroid administration [9,10,11]. Macrophages can differentiate into M1 macrophages, which are pro-inflammatory types, and M2 macrophages, which are tissue repair types—both M1 and M2 macrophages are reportedly involved in AE-IPF [9,10,12]. Although steroids act on various immune cells, the specific details remain unclear. One of the known immunological effects of steroids is their ability to inhibit monocyte/macrophage pro-inflammatory activation and induce the differentiation of anti-inflammatory cell phenotypes from naïve monocytes/macrophages [12,13]. A previous study on a lipopolysaccharide-induced acute lung injury model reported that methylprednisolone restored the M1 and M2 macrophage balance by shifting the macrophage phenotype from M1 to M2 [14]. Therefore, monitoring inflammatory macrophage immunity following steroid administration is essential for determining therapeutic responsiveness in AE-ILDs. However, the current early and convenient serum markers used to reflect the activity of immunocompetent cells, including macrophages in AE-ILDs, are insufficient.

Serum presepsin—the N-terminal fragment of the CD14 molecule—is predominantly expressed on the membranes of monocytes, macrophages, and dendritic cells [15,16]. Presepsin was originally recognized as an early biomarker for diagnosing sepsis and evaluating treatment efficacy in sepsis [15]. Notably, presepsin levels are elevated in infectious and non-infectious diseases, such as systemic lupus erythematosus (SLE) [17,18]. Furthermore, presepsin is produced during the phagocytosis of neutrophil extracellular traps caused by macrophages [17]. This suggests that presepsin may be useful as a biomarker, reflecting macrophage activity.

Although AE-ILD biomarkers are used in clinical practice, including C-reactive protein (CRP), lactate dehydrogenase (LDH), Krebs von den Lungen-6 (KL-6), and pulmonary surfactant protein D (SP-D), none of these classical markers alone determines the prognosis of AE-ILDs [3]. In this study, we focused on presepsin as a macrophage activation marker. We aimed to identify classical markers, including CRP, LDH, KL-6, and SP-D, that should be measured simultaneously with presepsin when assessing the therapeutic efficacy of steroid pulse therapy in AE-ILDs.

## 2. Materials and Methods

### 2.1. Study Design and Patients

All study procedures were conducted in accordance with the standards of the Ethical Review Board of the International University of Health and Welfare (approval number 20-Nr-101 [22 February 2021]) and conformed to the 1964 Declaration of Helsinki and its subsequent amendments or comparable ethical standards. The ethics committee waived the requirement for obtaining informed consent from patients, as this was a retrospective study limited to records collected as standard of care by respiratory physicians. All data were anonymized, ensuring the protection of patient privacy.

This single-center retrospective observational study involved 35 inpatients admitted to the International University of Health and Welfare Narita Hospital between May 2023 and March 2024 who received steroid pulse therapy (methylprednisolone at 1000 mg/day for 3 days) for AE-ILDs. Given that the pathology of AE has been reported in conditions other than IPF, we collectively refer to IPF, NSIP, CHP, and CTD-ILD as ILDs [5].

The following patients were excluded from the study: one with cancerous lymphangiopathy, one with acute interstitial pneumonia (AIP), one with pneumocystis pneumonia (PCP), two with acute respiratory distress syndrome (ARDS), two with diffuse alveolar hemorrhage (DAH), five with drug-induced lung injury, two without presepsin data, three whose prognoses were affected by malignancy, one without oxygen therapy at the initiation of steroid pulse therapy, and one whose prognosis was affected by heart failure. Patients who survived for 90 days after the initiation of steroid pulse therapy were assigned to the survival group (*n* = 9), and those who did not were assigned to the non-survival group (*n* = 7). Therefore, 16 patients were ultimately enrolled in this study.

### 2.2. Data Collection

All clinical data were extracted from the patients’ medical records. After the diagnosis of AE-ILDs, steroid pulse therapy was conducted. The day steroid pulse therapy was initiated was defined as day 1, and the samples corresponded to “before steroid pulse” samples; day 4 samples were defined as “after steroid pulse” samples. Laboratory test results on days 1 and 4 were extracted for comparison before and after steroid pulse therapy. Chest radiography or high-resolution CT findings were evaluated by more than two skilled pulmonologists. Chest radiography or high-resolution CT findings evaluated by the pulmonologists were extracted from day 0 or 1.

### 2.3. Evaluation of Respiratory Status Before and After Steroid Pulse Therapy

The indicators to assess the respiratory status, including SpO_2_, F_I_O_2_, and respiratory rate, were determined twice daily (the day shift and the night shift). For patients in poor clinical condition, these indices were measured four or more times per day. Among the daily respiratory status data, the worst SpO_2_/F_I_O_2_ values were extracted and used for statistics. The F_I_O_2_ of the nasal cannula was calculated by multiplying the amount of oxygen by 4 and adding 20 to the value. The F_I_O_2_ of a simple face mask was calculated by subtracting 1 from the administered amount of oxygen and multiplying the value by 0.1. The F_I_O_2_ of a reserved mask was calculated by multiplying by 0.1, with a maximum of 1.0 [19]. Patients who failed conventional oxygen therapy were initiated on high-flow oxygen therapy.

### 2.4. Statistical Analysis

We first compared the mean ± standard deviation (SD), median values, and interquartile ranges (IQRs) for continuous variables obtained between the survival and non-survival groups. Subsequently, the Kolmogorov–Smirnov (two-sided) and Shapiro–Wilk tests were used to assess normality, after which homoscedasticity was determined using the F-test. Between-group differences in the continuous variables were tested using the Mann–Whitney *U*-test, while paired comparisons were tested using the Wilcoxon signed-rank test. Fisher’s exact test was performed to evaluate the significance of differences between groups. Cut-off values were determined using receiver operating characteristic (ROC) curve analysis and the area under the curve (AUC). Higher AUC values indicated superior discriminatory ability as follows: excellent discrimination, 0.9 ≤ AUC; good discrimination, 0.80 ≤ AUC < 0.90; fair discrimination, 0.70 ≤ AUC < 0.80; poor discrimination, AUC < 0.70. For a diagnostic test to be meaningful, the AUC > 0.5 was required [20,21]. The outliers for each variable were identified using the Smirnov–Grubbs test. Correlations between the parameters were analyzed using Spearman’s correlation test and were evaluated as follows: strong (*r* = 0.7–1), moderate (*r* = 0.5–0.7), or weak (*r* = 0.3–0.5) [22]. All statistical analyses were performed using EZR (Saitama Medical Center, Jichi Medical University, Saitama, Japan), a graphical user interface for R, and a modified version of R Commander designed to add statistical functions frequently used in biostatistics [23]. All plots were created using GraphPad Prism version 10.4.1 for Windows (GraphPad Software, Boston, MA, USA).

## 3. Results

### 3.1. Patient Demographics and Clinical Characteristics

**Figure 1:**
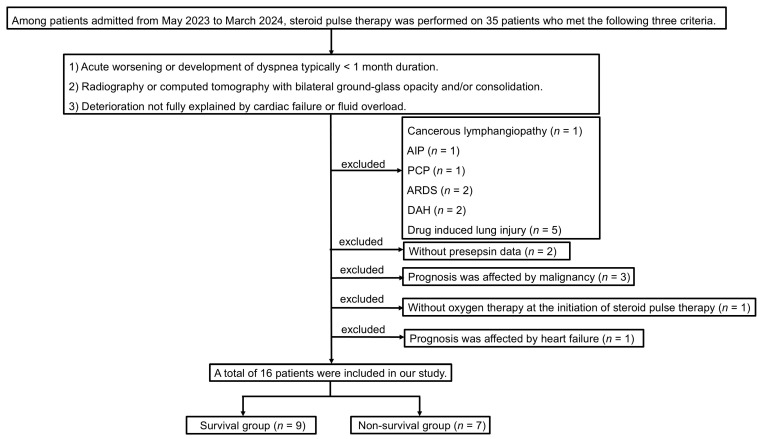
Study flowchart. The diagnosis of AE-ILDs was modified using the revised definition and diagnostic criteria for AE-IPF proposed by an international working group report as follows: (1) acute worsening or development of dyspnea, typically <1 month in duration; (2) CT with bilateral ground-glass opacity and/or consolidation; and (3) deterioration not fully explained by cardiac failure or fluid overload [4]. AE-ILDs, acute exacerbation of interstitial lung diseases; AIP, acute interstitial pneumonia; PCP, pneumocystis pneumonia; ARDS, acute respiratory distress syndrome; DAH, diffuse alveolar hemorrhage.

A total of 16 patients with AE-IPF, AE-NSIP, or RP-CTD-ILD were enrolled in this study (Figure 1). Table 1 shows their clinical characteristics. The univariate analysis revealed no significant differences between the groups.

### 3.2. Comparison of Presepsin Levels Before and After Steroid Pulse Therapy

**Figure 2:**
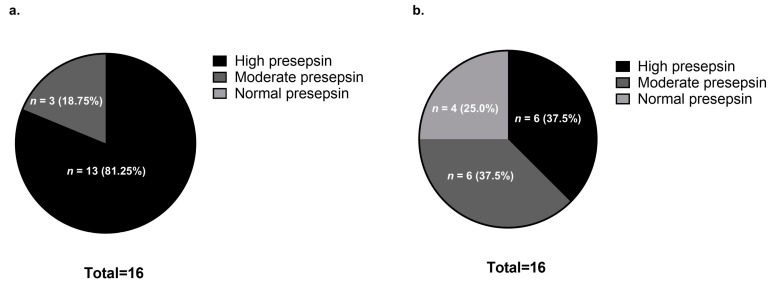
Comparison of presepsin levels before and after steroid pulse therapy: (**a**) Before steroid pulse therapy; (**b**) After steroid pulse therapy. Presepsin levels were classified into three types: (1) high presepsin levels: 470 pg/mL ≤ presepsin; (2) moderate presepsin levels: 294 pg/mL ≤ presepsin < 470 pg/mL; and (3) normal presepsin levels: presepsin < 294 pg/mL.

According to a previous report, the presepsin level in healthy individuals is 294 ± 121 pg/mL [24]. Additionally, the cut-off level for presepsin (upon admission) as a predictor of 30-day mortality for patients hospitalized with pneumonia is 470 pg/mL [25]. Based on these reports, we classified presepsin levels into three types: high presepsin (470 pg/mL or higher), moderate presepsin (294 pg/mL or more, but less than 470 pg/mL), and normal presepsin levels (less than 294 pg/mL). Based on this, the presepsin levels before steroid pulse therapy were abnormal in all 16 patients (100%), and high presepsin levels were observed in 81.25% (*n* = 13) of these patients (Figure 2a). However, the proportion of patients with elevated presepsin levels decreased to 37.5% (*n* = 6) after steroid pulse therapy, and the levels normalized in 25% (*n* = 4) of patients—all four of these patients were in the survival group (Figure 2b).

### 3.3. Clinical Course of Presepsin, CRP, LDH, and SP-D Levels Before and After Steroid Pulse Therapy

**Figure 3:**
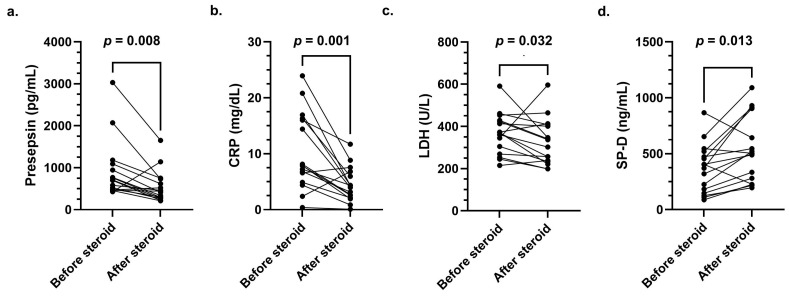
Comparison of presepsin, CRP, LDH, and SP-D levels before and after steroid pulse therapy using the Wilcoxon signed-rank test: (**a**) presepsin (*p* = 0.008); (**b**) CRP (*p* = 0.001); (**c**) LDH (*p* = 0.032); (**d**) SP-D (*p* = 0.013). CRP, C-reactive protein; LDH, lactate dehydrogenase; SP-D, pulmonary surfactant protein D.

Presepsin, CRP, LDH, and SP-D levels before and after steroid pulse therapy were compared for all 16 patients using the Wilcoxon signed-rank test. Significant differences in values before and after steroid pulse therapy were observed for all four markers (presepsin, *p* = 0.008; CRP, *p* = 0.001; LDH, *p* = 0.032; SP-D, *p* = 0.013, Figure 3).

### 3.4. Creation of Post-/Pre-Presepsin, CRP, LDH, and SP-D Ratios

To quantify the degree of improvement in the presepsin levels due to steroid pulse therapy, we created the post-/pre-presepsin ratio by dividing the presepsin level after (post) steroid pulse therapy by the level before (pre) steroid pulse therapy. The post-/pre-CRP, LDH, and SP-D ratios were similarly calculated.

### 3.5. Comparison of Post-/Pre-Presepsin, CRP, LDH, and SP-D Ratios in Survival and Non-Survival Groups

**Figure 4:**
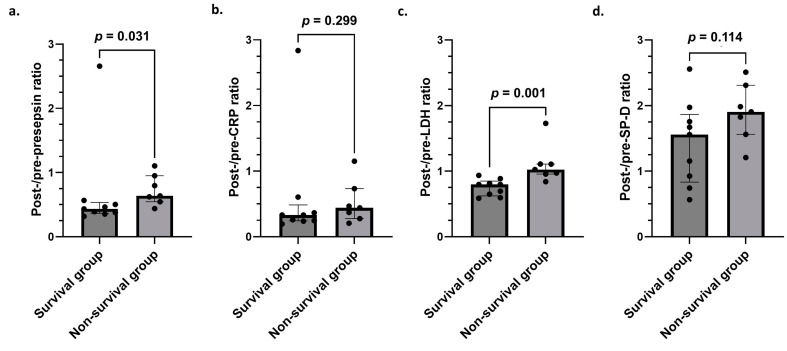
Comparison of post-/pre-presepsin, CRP, LDH, and SP-D ratios in survival and non-survival groups: (**a**) Post-/pre-presepsin ratio: 0.433 (0.378–0.504) vs. 0.636 (0.584–0.87), *p* = 0.031. (**b**) post-/pre-CRP ratio: 0.330 (0.246–0.36) vs. 0.441 (0.323–0.598), *p* = 0.299. (**c**) Post-/pre-LDH ratio: 0.797 (0.649–0.812) vs. 1.024 (0.963–1.107), *p* = 0.001. (**d**) Post-/pre-SP-D ratio: 1.558 (0.925–1.756) vs. 1.905 (1.693–2.146), *p* = 0.114. Mann–Whitney *U*-test. CRP, C-reactive protein; LDH, lactate dehydrogenase; SP-D, pulmonary surfactant protein D.

Comparisons between survival and non-survival groups regarding four ratios (post-/pre-presepsin, CRP, LDH, and SP-D ratios) were performed. As a result, post-/pre-presepsin and post-/pre-LDH ratios in the non-survival group were significantly higher than in the survival group. However, no significant differences were observed in the post-/pre-CRP and post-/pre-SP-D ratios between these two groups (Figure 4).

### 3.6. Creation and Performance of Post-/Pre-Presepsin–LDH Index as a Prognostic Marker for Non-Survival

**Figure 5:**
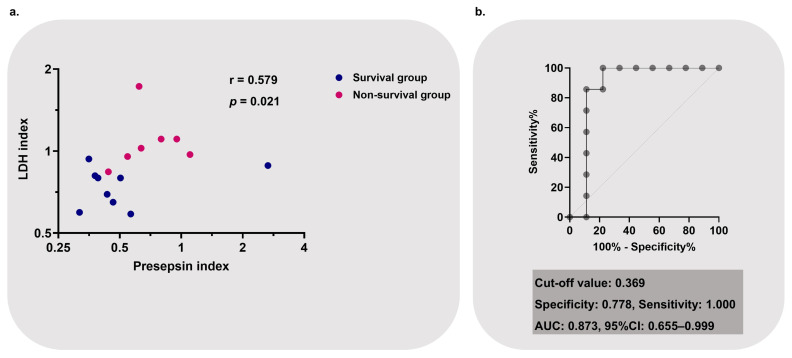
(**a**) Correlation between post-/pre-presepsin and LDH ratios; the relationship is statistically significant (r = 0.579; *p* = 0.021). (**b**) Performance of post-/pre-presepsin–LDH index as a prognostic marker for non-survival. Post-/pre-presepsin–LDH index: [post-/pre presepsin ratio] × [post-/pre LDH ratio]. Cut-off values for the post-/pre-presepsin–LDH index were calculated using ROC analysis. LDH, lactate dehydrogenase; ROC, receiver operating characteristic; AUC, area under the ROC curve; 95%CI, 95% confidence interval.

Figure 5a shows the correlation between the post-/pre-presepsin and LDH ratios. The post-/pre-presepsin and post-/pre-LDH ratios show a positive correlation. Based on this correlation, the value obtained from [post-/pre presepsin ratio] × [post-/pre LDH ratio] was defined as the post-/pre-presepsin–LDH index. Subsequently, we calculated the post-/pre presepsin–LDH index and performed receiver operating characteristic (ROC) analysis to evaluate the cut-off value for the non-survival group (Figure 5b). As a result, the AUC of the post-/pre-presepsin–LDH index showed good discrimination (AUC: 0.873; 95% confidence interval (CI): 0.655–0.999). When the cut-off value for the index was set to 0.369, the specificity was 77.8%, and the sensitivity was 100.0%.

### 3.7. Clinical Course After Steroid Pulse Therapy Based on the Post-/Pre-Presepsin–LDH Index

Table 2 presents the univariate analysis results comparing the general condition after steroid pulse therapy (day 4) and prognosis between the post-/pre-presepsin–LDH index < 0.369 and post-/pre-presepsin–LDH index ≥ 0.369 groups.

On day 4, we evaluated the patients’ general conditions using their laboratory data (CRP, LDH, SP-D, and D-dimer, quick sequential organ failure assessment (qSOFA) score), respiratory status (SpO_2_/F_I_O_2_, respiratory rate, and respiratory rate–oxygenation rate (ROX) index), and an oxygen device (conventional oxygen therapy (COT) and high-flow oxygen therapy (HFOT)) [26]. A qSOFA was performed to demonstrate that the results were not influenced by sepsis [27]. The SpO_2_/F_I_O_2_ and the ROX index in the post-/pre-presepsin–LDH index ≥ 0.369 group were significantly lower than in the post-/pre-presepsin–LDH index < 0.369 group. Furthermore, the proportion requiring COT was significantly higher in the post-/pre-presepsin–LDH index < 0.369 group than in the post-/pre-presepsin–LDH index ≥ 0.369 group; conversely, the proportion requiring HFOT was significantly higher in the post-/pre-presepsin–LDH index ≥ 0.369 group than in the post-/pre-presepsin–LDH index < 0.369 group.

Regarding prognosis, the proportion of patients who required additional treatment for AE-ILDs (a second instance of steroid pulse therapy and/or tacrolimus) and mortality were analyzed. The 60-day and 90-day mortality rates in the post-/pre-presepsin–LDH index ≥ 0.369 group were found to be significantly higher than in the post-/pre-presepsin–LDH index < 0.369 group (62.5% vs. 0.0%; *p* = 0.026, 75.0% vs. 12.5%; *p* = 0.041, respectively). No significant differences in additional treatment requirements were observed between the groups.

## 4. Discussion

Our study demonstrates several findings. First, the proportion of patients who showed high presepsin levels before steroid pulse therapy was high (81.25%). Furthermore, all four patients whose presepsin levels normalized after steroid pulse therapy were survivors. Second, the degree of improvement in the presepsin and LDH levels in the non-survival group was worse than that in the survival group. Third, in the ROC analysis, the post-/pre-presepsin–LDH index showed good discrimination as a prognostic marker for non-survival.

The high proportion of patients with elevated presepsin levels in this study suggests that those with AE-ILDs may demonstrate high levels of presepsin. As noted, the presepsin level in healthy individuals is 294 ± 121 pg/mL, and the cut-off value for presepsin in pneumonia patients is 470 pg/mL [24,25]. Additionally, the cut-off value of presepsin that maximizes the Youden index is 600–650 pg/mL [28]. Considering these data, the presepsin levels in this study’s patients were generally elevated. While there was no significant difference in the presepsin levels between the survival and non-survival groups before steroid pulse therapy, there was a significant difference between the two groups when comparing before and after steroid pulse therapy. Therefore, presepsin in AE-ILDs may represent an immunological pathway rather than simply reflecting disease severity. However, more clinical and basic data are needed to prove the immunological role of presepsin in AE-ILDs.

The present study shows that the post-/pre-presepsin ratio in the non-survival group was significantly higher than in the survival group, suggesting that the presepsin levels in the survival group may have been easier to reduce through steroid pulse therapy than in the non-survival group. Indeed, all patients whose presepsin levels normalized after steroid pulse therapy were in the survival group. Given these results, presepsin may be a useful indicator of treatment escalation in AE-ILDs.

The pathological conditions that support the relationship between AE-ILDs and presepsin are unknown. However, a previous study on sepsis reported that presepsin was released as a result of the phagocytosis of NETs caused by M1 macrophages [29]. Furthermore, our study shows that presepsin was released in the lungs, liver, and kidneys [29]. The findings that presepsin may be a marker of M1 macrophage activity and is produced by the lungs suggest its potential as an important biomarker in lung diseases involving M1 macrophage activity. However, this concept of the pathophysiology of AE-ILDs is merely speculation derived from a sepsis model. Further basic studies are required to prove that presepsin release through M1 macrophage activation is involved in the pathogenesis of AE-ILDs.

Elevated serum LDH levels reflect inflammation and tissue damage and are an essential marker in AE-ILDs [30]. Furthermore, LDH is a classical indicator of activity and severity in AE-IPF and is a useful biomarker for assessing treatment response, particularly in the acute phase [31]. Another report found that tracking LDH over time (2 weeks after hospitalization) may be useful for predicting prognosis in AE-IPF [32]. Our study also demonstrated the effectiveness of short-term follow-up for LDH in AE-ILDs. The post-/pre-LDH ratio in the non-survival group was significantly higher than in the survival group, similar to previous reports on AE-IPF. Combining LDH, which is an excellent short-term follow-up marker for AE-ILDs, with a more immune-specific marker (presepsin) may add value to the prognosis of AE-ILDs.

Since this study revealed that the post-/pre-presepsin ratio and the post-/pre-LDH ratio showed a positive correlation, we propose a potential marker that can simultaneously track the behavior of presepsin and LDH—the post-/pre-presepsin–LDH index. The analysis in this study shows that the post-/pre-presepsin–LDH index not only reflected respiratory status (ROX index and oxygen device) after steroid pulse therapy but also influenced mortality (as shown in Table 2). These results suggest the importance of simultaneously tracking presepsin and LDH during steroid pulse therapy in AE-ILDs.

Our study has several limitations. First, this was a single-center, retrospective study. Therefore, further clinical and basic studies are required to confirm our findings. Second, owing to the small sample size of the final cohort, multivariate analysis could not be performed. Hence, this pilot study may lead to basic and large-scale clinical research. Third, in addition to IPF, this study focused on NSIP, CHP, and CTD-ILD. The heterogeneity of AE-ILD pathophysiology should be noted in treatment [6]. However, just as ILDs have a variety of pathologies, the causes of AEs are diverse [5]. In clinical practice, there are many situations where it is impossible to determine whether an immunological mechanism is involved in an AE before steroid administration. Therefore, it is important to track the effects of steroids on AEs with common biomarkers, such as presepsin, regardless of the ILD phenotype. Fourth, presepsin is not commonly measured in clinical practice for AE-ILDs. However, when AE-ILDs are suspected, we routinely evaluate presepsin for infectious complications. Fifth, as shown in Figure 5a, only one patient’s post-/pre-presepsin ratio was an outlier. Indeed, this patient’s presepsin level before steroid pulse therapy was 429 pg/mL, increasing to 1140 pg/mL after steroid pulse therapy. However, this result may have been due to the storage conditions of the sample. Given that sample stirring is known to increase presepsin, we should have taken more care during sample preservation [33].

Altogether, these results suggest that presepsin may be a clinically useful marker to measure routinely to assess the therapeutic response of AE-ILDs. Furthermore, the clinical utility of the post-/pre-presepsin–LDH index needs to be verified in multicenter prospective cohort studies.

## 5. Conclusions

In conclusion, presepsin, which reflects macrophage activity, may serve as a prognostic and treatment-response biomarker in AE-ILDs. Furthermore, simultaneously tracking presepsin and LDH may serve as biomarkers for the early assessment of therapeutic response during steroid pulse therapy for these diseases. However, further prospective evaluation in heterogeneous ILD populations is required to validate these findings.

## Figures and Tables

**Table 1:** Patient demographic data and clinical characteristics.

Variable	Survival Group (*n* = 9)	Non-Survival Group (*n* = 7)	*p*-Value
Age (years)	71 (66–78)	72 (71–80)	0.458
Male sex (%)	6 (66.7%)	7 (100.0%)	0.212
Smoking history (%)	6 (66.7%)	4 (57.1%)	>0.999
Diabetes mellitus (%)	3 (33.3%)	3 (42.9%)	>0.999
Hypertension (%)	7 (80.0%)	4 (57.1%)	0.596
Malignancy (%)	1 (11.1%)	2 (28.6%)	0.550
AE-IPF (%)	5 (55.6%)	4 (57.1%)	>0.999
AE-NSIP (%)	1 (10.0%)	0 (0.0%)	>0.999
AE-CHP (%)	0 (0.0%)	0 (0.0%)	>0.999
RP-CTD-ILD (%)	3 (33.3%)	3 (42.9%)	>0.999
NT-proBNP	504 (273–1567)	2682 (428–2975)	0.606
Baseline treatment			
Prednisolone	1 (11.1%)	3 (42.9%)	0.262
Immunosuppressant	1 (11.1%)	1 (14.3%)	>0.999
Nintedanib	2 (22.2%)	3 (42.9%)	0.596
Pirfenidone	0 (0.0%)	1 (14.3%)	0.438

AE-IPF, acute exacerbation of idiopathic pulmonary fibrosis; AE-NSIP, acute exacerbation of idiopathic nonspecific interstitial pneumonia; AE-CHP, acute exacerbation of chronic hypersensitivity pneumonitis; RP-CTD-ILD, rapidly progressive connective tissue disease-associated interstitial lung disease; NT-proBNP, N-terminal natriuretic peptide.

**Table 2:** Clinical course after steroid pulse therapy based on the post-/pre-presepsin–LDH index.

Variable	Post-/Pre-Presepsin–LDH Index < 0.369 (*n* = 8)	Post-/Pre-Presepsin–LDH Index ≥ 0.369 (*n* = 8)	*p*-Value
After steroid pulse therapy (day 4)			
Laboratory data			
CRP (mg/dL)	2.64 (1.67–6.17)	4.34 (3.77–5.17)	0.270
SP-D (ng/mL)	511 (228–638)	497 (321–708)	>0.999
D-dimer (μg/mL)	2.93 (1.65–7.58)	3.16 (2.38–6.96)	0.798
qSOFA (score)			
0	5 (62.5%)	1 (12.5%)	0.119
1	3 (37.5%)	7 (87.5%)	0.119
2	0 (0.0%)	0 (0.0%)	>0.999
3	0 (0.0%)	0 (0.0%)	>0.999
Respiratory condition			
SpO_2_/F_I_O_2_	339 (283–394)	204 (116–303)	0.031
Respiratory rate (breaths/minute)	18 (18–20)	24 (19–26)	0.309
ROX index	18.85 (14.93–20.24)	8.19 (4.67–16.44)	0.040
Oxygen device			
Conventional oxygen therapy (%)	8 (100.0%)	3 (37.5%)	0.026
High-flow oxygen therapy (%)	0 (0.0%)	5 (62.5%)	0.026
Prognosis			
Additional treatment requirement			
Second steroid pulse therapy	1 (12.5%)	4 (50.0%)	0.282
Tacrolimus	2 (25.0%)	3 (37.5%)	>0.999
Mortality			
30-day mortality	0 (0.0%)	4 (50.0%)	0.077
60-day mortality	0 (0.0%)	5 (62.5%)	0.026
90-day mortality	1 (12.5%)	6 (75.0%)	0.041

CRP, C-reactive protein; LDH, lactate dehydrogenase; qSOFA, quick sequential organ failure assessment; ROX index, respiratory rate–oxygenation rate; SP-D, pulmonary surfactant protein D.

## Data Availability

The data supporting the findings of this study are available from the corresponding author upon reasonable request.
